# Octahedral Rhenium Cluster Complexes with 1,2-Bis(4-pyridyl)ethylene and 1,3-Bis(4-pyridyl)propane as Apical Ligands

**DOI:** 10.3390/molecules27227874

**Published:** 2022-11-15

**Authors:** Anton A. Ulantikov, Konstantin A. Brylev, Taisiya S. Sukhikh, Yuri V. Mironov, Viktoria K. Muravieva, Yakov M. Gayfulin

**Affiliations:** Nikolaev Institute of Inorganic Chemistry SB RAS, 3, Academician Lavrentiev Ave., 630090 Novosibirsk, Russia

**Keywords:** rhenium, cluster, pyridine ligand, DFT calculations, electrochemistry, luminescence

## Abstract

A series of eight new octahedral rhenium cluster complexes with the general formula *trans*-[{Re_6_Q_8_}L_4_X_2_] (Q = S or Se; L = 1,2-Bis(4-pyridyl)ethylene (bpe) or 1,3-Bis(4-pyridyl)propane (bpp); X = Cl or Br) was synthesized and investigated. While bpe is a ligand with a conjugated aromatic system, bpp represents a molecule of opposite type and has independent aromatic systems of the two pyridine rings. It was shown that this difference in the electronic structure of the ligands has a fundamental effect on the electronic structure, electrochemical and luminescent properties of the corresponding cluster complexes. Specifically, the [{Re_6_Q_8_}(bpe)_4_X_2_] complexes in solutions show multiple quasi-reversible electrochemical transitions associated with reduction of the organic ligands. At the same time, the *trans*-[{Re_6_Q_8_}(bpp)_4_X_2_] complexes show multielectron quasi-irreversible reduction processes, which correlate with the mixed character of the lowest unoccupied molecular orbitals of these complexes. All the obtained new compounds exhibit red photoluminescence. The photophysical parameters (emission lifetimes and quantum yields) measured for the bpp complexes exceed those revealed for bpe complexes by more than an order of magnitude.

## 1. Introduction

Octahedral chalcogenide cluster complexes formed by transition metals of groups 5–7 have been studied for more than two decades [1,2,3]. Coordination of various organic ligands allows change in the functional properties of the complexes, such as luminescence, reversible electrochemical transitions and ability to assemble polymeric and oligomeric compounds [4,5,6,7,8]. A fairly large number of octahedral rhenium clusters with N, P, and S-donor ligands are known [9,10]. However, a systematic study of the influence of the type, number, and coordination isomerism of organic ligands on the spectroscopic and electrochemical properties of the hexarhenium clusters was carried out to a limited extent. To date, the most detailed research in this area was carried out for compounds with the general formula [{Re_6_S_8_}(L)_2_Cl_4_]^2-^, where L = pyrazine (pz), 4,4′-bpyridine (bpy), 4-cyanopyridine (cpy), pyridine (py), 4-methylpyridine (mpy), 4-(dimethylamino)pyridine (dmap) [11,12]. In particular, these complexes were studied by cyclic voltammetry and differential pulse voltammetry. It was shown that the pK_a_ value of an organic base correlates with the shift of potential of the oxidation process belonging to the cluster core. Thus, in the series dmap–mpy–py–bpy–cpy–pz, the values of the potentials of the Re^III^_6_/Re^III^_5_Re^IV^ process range from +0.68 to +0.86 V correlating linearly with the decrease of pK_a_. In addition, the influence of the isomerism on the oxidation potential was shown to be negligible. It was also shown that, upon coordination of redox active organic molecules such as pz, cpy and bpy, ligand-centered reduction processes appeared on cyclic voltammetry (CV) curves of corresponding compounds. The position of these processes largely depends on the nature of the coordinated molecule. The anodic shift of the first reduction potential of organic ligands in comparison with corresponding free molecules in solution was 0.76, 0.60, and 0.45 V for pz, cpy, and bpy, respectively, which also correlates with the pK_a_ values. Investigation of the series of clusters coordinated by bpy ligands have shown that an increase of the number of organic ligands coordinated to the cluster core causes the process of core oxidation to lose reversibility together with an increase of the anodic shift of the ligand-centered reduction process. Thus, the measured anodic shifts were determined to be 0.44, 0.69, and 0.82 V for [Re_6_S_8_Cl_4_(bpy)_2_]^2−^, [Re_6_S_8_Cl_3_(bpy)_3_]^−^, and [Re_6_S_8_Cl_2_(bpy)_4_], respectively [13].

Based on the electrochemical properties of clusters with organic ligands, it was suggested that π*-orbitals of redox-active ligands make a significant contribution to the LUMO of the complexes. Subsequently, quantum chemical calculations were carried out for these compounds, and this hypothesis was confirmed [13,14]. It was shown that the energy of ligand π*-orbitals decreases upon coordination, and they are embedded between the metal-centered orbitals of the cluster core, becoming available for filling with electrons. Thus, the electron-donor nature of N-heteroaromatic ligands is directly related to the ability of their MO to be incorporated into the electronic structure of the metal complex which affects spectroscopic and photophysical properties of the cluster complexes by changing the HOMO-LUMO gap.

**Figure 1:**
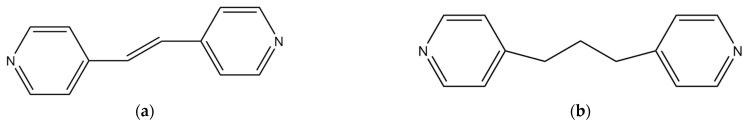
Molecules of 1,2-Bis(4-pyridyl)ethylene (bpe, **a**) and 1,3-Bis(4-pyridyl)propane (bpp, **b**).

We continued to study the mutual influence of coordinated N-heteroaromatic ligands and octahedral rhenium cluster complexes on the properties of the resulting compounds. Here we present two series of new compounds with the general formula *trans*-[Re_6_Q_8_L_4_X_2_] (Q = S or Se; X = Cl or Br; L = 1,2-Bis(4-pyridyl)ethylene (bpe) or 1,3-Bis(4-pyridyl)propane (bpp), Figure 1). Like 4,4′-bpyridine, bpe is a ligand with a conjugated π-system. On the contrary, bpp is an example of a ligand with separate electronic subsystems of aromatic rings. It has been shown that this difference has a fundamental effect on electrochemical behavior and spectroscopic characteristics of the new cluster complexes.

## 2. Experimental

### 2.1. Materials and Methods

The initial compounds Cs_n_[Re_6_Q_8_X_6_]·2H_2_O (Q = S, *n* = 4; Q = Se, *n* = 3; X = Cl or Br) were prepared according to the known procedure [15]. Other reagents and solvents were used without additional purification.

Elemental (CHNS) analysis was carried out on an EuroVector EA3000 analyzer. Energy dispersive spectroscopy study was performed on a Hitachi TM-3000 electron microscope with a Bruker Nano EDS analyzer. IR spectra of powder samples in KBr pellets were recorded on a Bruker Scimitar FTS 2000 spectrometer in the range of 4000–375 cm^−1^. Excitation and emission spectra of powdered samples were recorded on a Horiba Jobin Yvon Fluorolog 3 spectrometer with a 450 W xenon arc lamp, a cooled PC177CE-010 photodetector, and a PMT R2658 double grating monochromator. The samples were placed in a teflon cell and covered with a quartz glass. The spectra were corrected for source intensity and emission spectral response by standard correction curves. The quantum yields were determined using a Quanta-φ integrating sphere (Horiba Jobin Yvon). The luminescence lifetime was measured using a NanoLED photon-counting detector with pulsed light sources. All studies of luminescence properties were carried out at 298 K. Cyclic voltammetry was carried out on an Elins P-20X8 voltammetry analyzer using a three-electrode scheme with a glassy carbon (GC) working electrode, a Pt auxiliary electrode, and a Ag/AgCl/3.5 M KCl reference electrode. Investigations were carried out for 1.5 × 10^–3^ M solutions of cluster salts **1**–**3**, **5**–**8** in 0.1 M Bu_4_NBF_4_ in DMSO under Ar atmosphere. The registered value of E_1/2_ for the Fc^0/+^ couple was 0.130 V in the same conditions.

### 2.2. Preparation of Trans-[Re_6_S_8_(bpe)_4_Cl_2_] (***1***)

Cs_4_[Re_6_S_8_Cl_6_]·2H_2_O (0.200 g, 0.09 mmol) and 1,2-bis(4-pyridyl)ethylene (bpe, 0.200 g, 1.09 mmol) were loaded into a glass ampule. The ampule was sealed, heated to 200 °C in a furnace, and kept at this temperature for 24 h. After cooling, the resulting reaction mixture contained orange crystals of [Re_6_S_8_(bpe)_4_Cl_2_]·2.75bpe (**1·solv**). To remove the excess of bpe and unreacted cluster precursor, the reaction mixture was washed successively with boiling EtOH (3 portions of 15 mL) and boiling water (3 portions of 15 mL) in a glass beaker with stirring. The insoluble residue of compound **1** was separated by centrifugation and dried in air. Yield (calculated on the precursor Cs_4_[Re_6_S_8_Cl_6_]·2H_2_O): 0.188 g (93%). FT-IR (KBr, cm^−1^): 1,2-Bis(4-pyridyl)ethylene (bpe): 551, 667, 829, 908, 972, 1028, 1062, 1105, 1203, 1220, 1251, 1296, 1340, 1373, 1427, 1506, 1541, 1558, 1610, 1716, 2461, 2835, 2910, 3034; ν(ReS) 416. EDS (pellet): Cl:Re:S = 2.2:6,0:8.4. Elemental analysis calculated (%) for C_48_H_40_N_8_Re_6_S_8_Cl_2_: C 26.47, H 1.85, N 5.14, S 11.76. Found: C 26.48, H 1.82, N 5.13, S 11.78. ^1^H NMR ((CD_3_)_2_SO, 25 °C): δ = 7.67 (s, 8H; -CH-), δ = 7.72 (d, 8H; H_m_), 7.95 (d, 8H, H_m_), 8.74 (d, 8H, H_o_), 9.34 ppm (d, 8H, H_o_).

### 2.3. Preparation of Trans-[Re_6_S_8_(bpe)_4_Br_2_] (***2***)

Compound **2** was prepared by a procedure similar to that for **1** using Cs_4_[Re_6_S_8_Br_6_]·2H_2_O (0.200 g, 0.08 mmol) as a precursor. Orange crystals of [Re_6_S_8_(bpe)_4_Cl_2_]·3bpe (**2·solv**) were obtained from the melt before washing. Yield (calculated on the precursor Cs_4_[Re_6_S_8_Br_6_]·2H_2_O): 0.172 g (92%). FT-IR (KBr, cm^−1^): bpe: 551, 669, 829, 910, 977, 1026, 1062, 1103, 1203, 1222, 1251, 1296, 1338, 1375, 1425, 1496, 1541, 1558, 1610, 1716, 2457, 2825, 2902, 3034; ν(ReS) 418. EDS (pellet): Br:Re:S = 2.4:6.0:8.0. Elemental analysis calculated (%) for C_48_H_40_N_8_Re_6_S_8_Br_2_: C 25.44, H 1.78, N 4.95, S 11.29. Found: C 25.47, H 1.82, N 4.98, S 11.26%. ^1^H NMR ((CD_3_)_2_SO, 25 °C): δ = 7.67 (s, 8H; CH), δ = 7.71 (d, 8H; H_m_), 7.94 (d, 8H, H_m_), 8.72 (d, 8H, H_o_), 9.31 ppm (d, 8H, H_o_).

### 2.4. Preparation of Trans-[Re_6_Se_8_(bpe)_4_Cl_2_] (***3***)

Compound **3** was prepared by a procedure similar to that for **1** using Cs_3_[Re_6_Se_8_Cl_6_]·2H_2_O (0.200 g, 0.08 mmol) as a precursor. Yield (calculated on the precursor Cs_3_[Re_6_Se_8_Cl_6_]·2H_2_O): 0.190 g (94%). FT-IR (KBr, cm^−1^): bpe: 549, 665, 738, 829, 908, 966, 1024, 1060, 1103, 1203, 1219, 1251, 1296, 1338, 1375, 1419, 1506, 1541, 1558, 1608, 1716, 2472, 2835, 2922, 3055. EDS (pellet): Cl:Re:Se = 2.3:6.0:8.1. Elemental analysis calculated (%) for C_48_H_40_N_8_Re_6_Se_8_Cl_2_: C 22.51, H 1.57, N 4.38. Found: C 22.54, H 1.60, N 4.36%. ^1^H NMR ((CD_3_)_2_SO, 25 °C): δ = 7.67 (s, 8H; -CH-), δ = 7.72 (d, 8H; H_m_), 7.90 (d, 8H, H_m_), 8.71 (d, 8H, H_o_), 9.29 ppm (d, 8H, H_o_).

### 2.5. Preparation of Trans-[Re_6_Se_8_(bpe)_4_Br_2_] (***4***)

Compound **4** was prepared by a procedure similar to that for **1** using Cs_3_[Re_6_Se_8_Br_6_]·2H_2_O (0.200 g, 0.08 mmol) as a precursor. Yield (calculated on the precursor Cs_3_[Re_6_Se_8_Br_6_]·2H_2_O): 0.173 g (92%). FT-IR (KBr, cm^−1^): bpe: 547, 667, 740, 827, 910, 966, 1024, 1062, 1107, 1203, 1219, 1251, 1340, 1375, 1419, 1506, 1541, 1558, 1602, 1716, 2380, 2835, 2929, 3064. EDS (pellet): Br:Re:Se = 2.2:6.0:8.3. Elemental analysis calculated (%) for C_48_H_40_N_8_Re_6_Se_8_Br_2_: C 21.76, H 1.52, N 4.23. Found: C 21.80, H 1.51, N 4.26%.

### 2.6. Preparation of Trans-[Re_6_S_8_(bpp)_4_Cl_2_] (***5***)

A solution of Cs_4_[Re_6_S_8_Cl_6_]·2H_2_O (0.200 g, 0.09 mmol) and 1,3-bis(4-pyridyl)propane (bpp, 0.2 g, 1.01 mmol) in 3 mL of water was loaded into a glass ampule. The ampule was sealed, heated to 160 °C in a furnace, kept at this temperature for 24 h, and then cooled down at a natural rate. The target product settled to the bottom in the form of a red oil. Water was removed and the oil was dissolved in 10 mL of acetonitrile. After that, diethyl ether was added for deposition of a powder. The powder was washed with diethyl ether (3 portions of 15 mL) and dried in air. Yield (calculated on the precursor Cs_4_[Re_6_S_8_Cl_6_]·2H_2_O): 0.208 g (98 %). FT-IR (KBr, cm^−1^): 1,3-bis(4-pyridyl)propane (bpp): 503, 580, 617, 650, 667, 740, 813, 912, 1001, 1026, 1062, 1109, 1207, 1226, 1340, 1373, 1425, 1456, 1504, 1541, 1558, 1614, 1637, 1716, 2127, 2617, 2854, 2926, 3066; ν(ReS) 418. EDS (pellet): Cl:Re:S = 2.3:6.0:8.4. Elemental analysis calculated (%) for C_52_H_56_N_8_Re_6_S_8_Cl_2_: C 27.86, H 2.52, N 5.00, S 11.41. Found: C 27.88, H 2.49, N 5.04, S 11.44%. ^1^H NMR ((CD_3_)_2_SO, 25 °C): δ = 3.44 (s, 24H; -CH_2_-), δ = 7.36 (d, 8H; H_m_), 7.64 (d, 8H, H_m_), 8.59 (d, 8H, H_o_), 9.48 ppm (d, 8H, H_o_).

### 2.7. Preparation of Trans-[Re_6_S_8_(bpp)_4_Br_2_] (***6***)

Compound **6** was prepared by a procedure similar to that for **5** using Cs_4_[Re_6_S_8_Br_6_]·2H_2_O (0.2 g, 0.08 mmol) as a precursor. Yield (calculated on the precursor Cs_4_[Re_6_S_8_Br_6_]·2H_2_O): 0.182 g (95 %). FT-IR (KBr, cm^−1^): bpp: 5010, 584, 657, 651, 667, 742, 814, 912, 1000, 1024, 1062, 1110, 1207, 1226, 1337, 1376, 1425, 1445, 1510, 1541, 1559, 1611, 1644, 1716, 2135, 2622, 2854, 2926, 3062; ν(ReS) 418. EDS (pellet): Br:Re:S = 2.0:6.0:8.1. Elemental analysis calculated (%) for C_52_H_56_N_8_Re_6_S_8_Br_2_: C 26.81, H 2.42, N 4.81, S 10.99. Found: C 26.77, H 2.40, N 4.85, S 11.02%. ^1^H NMR ((CD_3_)_2_SO, 25 °C): δ = 3.43 (s, 24H; -CH_2_-), δ = 7.36 (d, 8H; H_m_), 7.63 (d, 8H, H_m_), 8.58 (d, 8H, H_o_), 9.43 ppm (d, 8H, H_o_).

### 2.8. Preparation of Trans-[Re_6_Se_8_(bpp)_4_Cl_2_] (***7***)

Compound **7** was prepared by a procedure similar to that for **5** using Cs_3_[Re_6_Se_8_Cl_6_]·2H_2_O (0.2 g, 0.08 mmol) as a precursor. Yield (calculated on the precursor Cs_3_[Re_6_Se_8_Cl_6_]·2H_2_O): 0.198 g (96%). FT-IR (KBr, cm^−1^): bpp: 504, 585, 619, 655, 666, 747, 812, 912, 1001, 1025, 1072, 1112, 1208, 1233, 1342, 1370, 1425, 1454, 1508, 1543, 1547, 1609, 1635, 1720, 2129, 2617, 2850, 2933, 3056. EDS (pellet): Cl:Re:Se = 2.3:6.0:8.2. Elemental analysis calculated (%) for C_52_H_56_N_8_Re_6_Se_8_Cl_2_: C 23.79, H 2.15, N 4.27. Found: C 23.84, H 2.11, N 4.28%. ^1^H NMR ((CD_3_)_2_SO, 25 °C): δ = 3.43 (s, 24H; -CH_2_-), δ = 7.36 (d, 8H; H_m_), 7.58 (d, 8H, H_m_), 8.59 (d, 8H, H_o_), 9.38 ppm (d, 8H, H_o_).

### 2.9. Preparation of Trans-[Re_6_Se_8_(bpp)_4_Br_2_] (***8***)

Compound **8** was prepared by a procedure similar to that for **5** using Cs_3_[Re_6_Se_8_Br_6_]·2H_2_O (0.2 g, 0.08 mmol) as a precursor. Yield (calculated on the precursor Cs_3_[Re_6_Se_8_Br_6_]·2H_2_O): 0.183 g (95%). FT-IR (KBr, cm^−1^): bpp: 511, 577, 619, 649, 658, 744, 808, 919, 1010, 1021, 1066, 1107, 1206, 1225, 1336, 1375, 1428, 1452, 1501, 1540, 1539, 1617, 1632, 1718, 2117, 2599, 2844, 2914, 3066. EDS (pellet): Br:Re:Se = 2.1:6.0:8.1. Elemental analysis calculated (%) for C_52_H_56_N_8_Re_6_Se_8_Br_2_: C 23.01, H 2.08, N 4.13. Found: C 22.98, H 2.10, N 4.11%. ^1^H NMR ((CD_3_)_2_SO, 25 °C): δ = 3.42 (s, 24H; CH_2_), δ = 7.34 (d, 8H; H_m_), 7.58 (d, 8H, H_m_), 8.57 (d, 8H, H_o_), 9,36 ppm (d, 8H, H_o_).

### 2.10. The X-ray Diffraction (XRD) Analysis

The XRD analysis of **1** and **2** was performed according to the standard procedure on a Bruker Venture diffractometer with a CMOS PHOTON III detector and a IμS 3.0 source (Montel mirrors, Mo*K*_α_ radiation). The reflection intensities were measured by φ- and ω-scanning of narrow (0.5°) frames. The absorption correction was calculated with the SADABS program [16]. The structures were solved using the SHELXT program [17] and refined with the SHELXL program [18] using the Olex2 interface [19]. The positions of H atoms were calculated geometrically and refined using a riding model. All data were obtained on the equipment of the XRD Facility of NIIC SB RAS. The crystallographic data and details of the structure refinements are summarized in Table S1 (Supplementary Material). The XRD data were deposited with the Cambridge Crystallographic Data Centre (numbers 2212901 and 2212902) and can be requested from the authors or at http://www.ccdc.cam.ac.uk/conts/retrieving.html (accessed on 14 October 2022).

### 2.11. Calculation Details

Density functional theory (DFT) calculations were carried out for the *trans*-[Re_6_S_8_(bpe)_4_Cl_2_] and *trans*-[Re_6_S_8_(bpp)_4_Cl_2_] molecular cluster complexes in the ADF2021 program package [20,21]. Geometric parameters for the clusters in *C*_1_ symmetry were optimized with VWN + S12g dispersion-corrected density functional [22,23,24] and all-electron TZ2P basis set [25]. The calculated vibrational spectra contain no imaginary frequencies. Single-point calculations of bonding energies and molecular orbitals with geometries from VWN + S12g/TZ2P level of theory were carried out with a dispersion-corrected hybrid density functional S12h and an all-electron TZ2P basis set [24]. Additionally, optimized geometry, bonding energies, and molecular orbitals were calculated for the [Re_6_S_8_Cl_6_]^4–^ anion by the same computational procedure. DMSO environment for all clusters was simulated by COSMO method [26,27]. The zero-order regular approximation (ZORA) was used in all calculations to take into account scalar relativistic effects [28]. Atomic coordinates of the clusters with the optimized geometry are presented in Tables S2 and S3 (Supplementary Material).

## 3. Results and Discussion

### 3.1. Synthesis

There are two common approaches to the preparation of octahedral rhenium cluster complexes with organic apical ligands. The first approach is the reaction of the cluster in a solvent or solvent mixture with the organic ligand excess. The main disadvantage of this method is the possible formation of products with various numbers of substituted ligands and their isomers. Isolation of these products as individual compounds often requires complex separation procedures, and the yield of products decreases as the degree of substitution increases. The second approach is the reaction of the cluster with the proligand melt without the addition of a solvent. This synthetic procedure allows one to shift the reaction to the most thermodynamically stable product and obtain it with the yield close to quantitative. However, this synthetic approach limits the range of possible organic ligands to the compounds melting without decomposition. This approach was successfully applied earlier for the preparation of clusters with the formula trans-[Re_6_Q_8_(L)_4_X_4_] (Q = S, Se; X = Cl, Br; L = 4,4′-bpyridine [14], phenylpyridine [29]). Here, cluster complexes with bpe were obtained under similar conditions.

The synthesis of compounds **1**–**4** proceeded at a temperature of 200 °C with the formation of crystalline compounds containing neutral clusters *trans*-[Re_6_Q_8_(bpe)_4_X_2_] as well as several crystallization bpe molecules. Compounds become X-ray amorphous after purification from unreacted precursor and crystallization bpe molecules; however, they have the composition [Re_6_Q_8_(bpe)_4_X_2_] according to elemental analysis. Similar to the compounds *trans*-[Re_6_Q_8_(bpy)_4_X_2_] obtained earlier, the cluster complexes with bpe are soluble in DMSO, except for the trans-[Re_6_Se_8_(bpe)_4_Br_2_] cluster. This made it possible to obtain NMR spectroscopy data. As expected, ^1^H NMR spectra in (CD_3_)_2_SO demonstrate a shift of the proton signals belonging to the coordinated ring towards the strong field. The proton signals of the ethylene bridge are also shifted similarly (Figure S1a, Supplementary Material). NMR data confirm the absence of non-coordinated bpe in compounds **1–4**.

Compounds [Re_6_Q_8_(bpp)_4_X_2_] (**5**–**8**) were obtained by hydrothermal synthesis with a large excess of organic ligand at a temperature of 160 °C since the reaction in the melt of bpp led to formation of unidentified brown products. As a result of hydrothermal synthesis, the red X-ray amorphous compounds soluble in organic solvents were isolated. According to elemental analysis data, the compounds have the composition [Re_6_Q_8_(bpp)_4_X_2_] (Q = S, Se; X = Cl, Br). Unfortunately, our attempts to obtain crystals suitable for single crystal XRD failed. However, the compounds are highly soluble in DMSO, and their structure has been proven by NMR spectroscopy. As one can see in Figure S1b, the position of the proton signals belonging to the coordinated aromatic ring is shifted towards the strong field. Moreover, the signal shift magnitude of the protons that are closer to the coordinated nitrogen atom (positions 2 and 6) is noticeably higher than that of protons in positions 3 and 5. The rest of the signals coincide with the signals of the free ligand. It was shown earlier for the disubstituted clusters [Re_6_S_8_Cl_4_L_2_]^2–^ that ortho proton signals of the pyridine derivatives in the *trans*-isomer appear at higher magnetic fields than in the case of the *cis*-isomer [8]. Since we observed only one set of signals for each [Re_6_Q_8_X_2_L_4_] cluster and the chemical shifts of the ortho proton signals of [Re_6_Q_8_X_2_(bpp)_4_] clusters are close to ones for structurally characterized [Re_6_Q_8_X_2_(bpe)_4_] clusters, we may propose that *trans*-isomers of [Re_6_Q_8_X_2_(bpp)_4_] clusters were formed selectively. 

### 3.2. Crystal Structures

Octahedral rhenium clusters coordinated by N-donor aromatic ligands are widely presented in the literature. These ligands form numerous weak interactions, which often lead to very stable and insoluble compounds [30,31]. It is interesting to note that most structures of octahedral rhenium cluster complexes with organic ligands contain solvate organic molecules [32]. We have found that these solvate molecules can be removed by washing the compounds with ethanol. This leads to the formation of X-ray amorphous substances [14,29,33].

**Figure 2:**
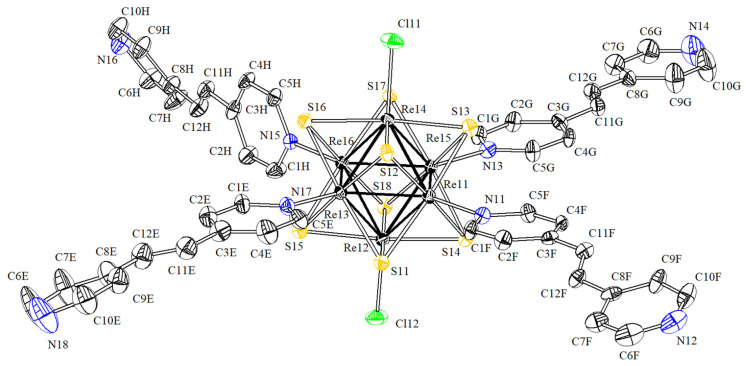
Molecular cluster [Re_6_S_8_(bpe)_4_Cl_2_] in the structure of compound **1·solv**.

Compound **1·solv** crystallizes in triclinic crystal system, space group *P*$\bar{1}$. The asymmetric unit contains two cluster fragments [Re_6_S_8_(bpe)_4_Cl_2_], six solvate bpe molecules and half of the solvate pro-ligand molecule: one pyridine ring and one CH group belonging to the alkene bridge. Four bpe molecules are coordinated to the rhenium atoms of both cluster fragments in the equatorial plane. Re–N bonds have lengths 2.178(6)–2.195(6) Å (Figure 2). Two chloride ions are coordinated in the *trans*-position and form Re–Cl bonds with lengths of 2.4011(19)–2.4134(19) Å.

**Figure 3:**
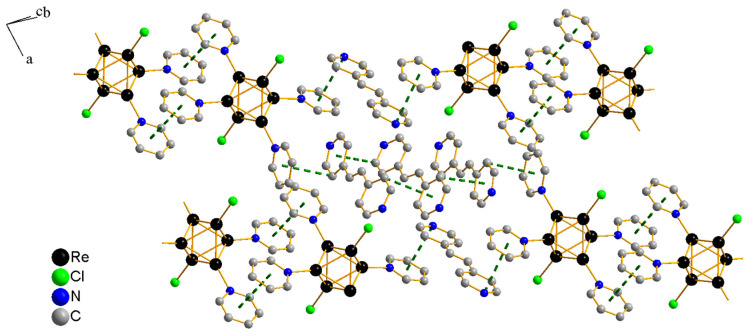
Packing fragment of cluster fragments in the structure of compound **1·solv**. Solvate bpe molecules and parts of the ligands not involved in binding interactions, as well as µ_3_-S ligands, are not shown.

The ligands of neighboring cluster fragments overlap and form π-interactions with distances between aromatic rings centroids of 3.78 Å. Short contacts are also formed between µ_3_-S ligands with a length of 3.30 Å (Figure S2, Supplementary Material). The packing of compound **1·solv** is formed by these dimeric fragments linked with solvate bpe molecules due to C–H…π interactions with lengths of 3.67–3.40 Å. The centroids of the metal clusters are located on the (111) axis. Additionally, these fragments are interconnected through one solvate molecule using π-stacking, with an N-centroid distance of 3.53–3.65 Å. Thus, they form layers of metal clusters in the (011) plane (Figure 3). The relative orientation of ligands within one cluster fragment is determined by packing. One of the cluster fragments has three bpe ligands, in which both pyridyl rings are located almost parallel to the equatorial plane of the cluster core. The remaining ligand is located at an angle of 82° to this plane. In another cluster fragment, only two ligands are located parallel to the equatorial plane of the metal cluster. The other two form angles of 87° and 23° with this plane. Additional solvate bpe molecules are located in the interlayer space, occupying the entire available volume between cluster fragments.

**Figure 4:**
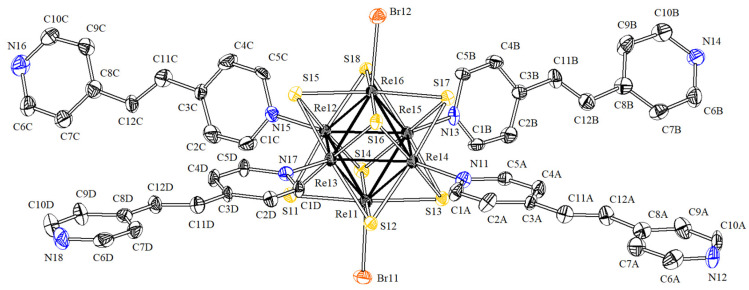
Molecular cluster [Re_6_S_8_(bpe)_4_Br_2_] in the structure of compound **2·solv**.

The compound **2·solv** crystallizes in the triclinic crystal system, space group P$\bar{1}$. The asymmetric fragment contains one cluster [Re_6_S_8_(bpe)_4_Br_2_], two solvate bpe molecules, and three (NC_5_H_5_CH) fragments, which generate solvate bpe molecules through the inversion center located in the center of the alkene bridge. The alkyl bridges are disordered over two positions in two of these molecules. Four bpe molecules are coordinated to rhenium atoms in the equatorial plane. Re–N bonds have lengths 2.174(6)–2.194(6) Å. Also, two bromine ions are coordinated in the trans position, forming Re–Br bonds with lengths of 2.560(1) Å and 2.565(1) Å (Figure 4). In this compound, the ligands of adjacent cluster fragments also overlap and form a network of π-interactions with distances between aromatic rings centroids of 3.78 Å. Additionally, short contacts are formed between the µ_3_-S ligands with a length of 3.30 Å (Figure S3, Supplementary Material).

**Figure 5:**
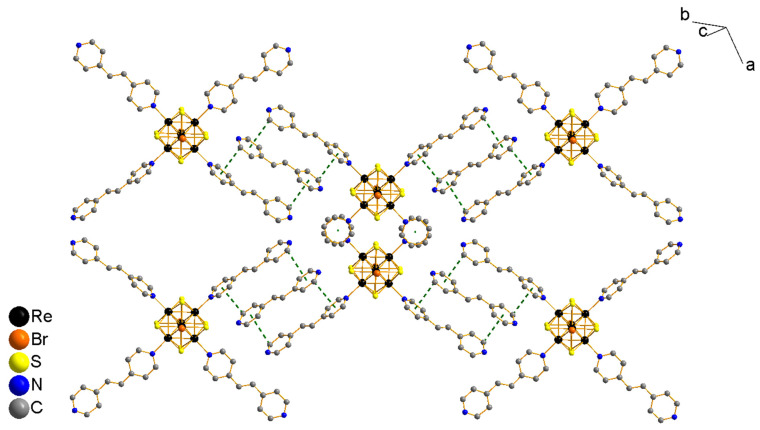
Packing fragment of cluster fragments in the structure of compound **1·solv**. Solvate bpe molecules and parts of the ligands that do not form weak interactions in this plane are not shown.

The packing of this compound is formed in such a way that a pair of ligands coordinated in the *cis*-position parallel to the equatorial plane of the cluster core participates in the formation of dimeric fragments where cluster units are bonded by π-stacking between pairs of the aromatic rings closest to the cluster core (Figure S3). The other pair of ligands, which are located almost perpendicular to this plane, forms layers through π-stacking with the bpe solvate molecule. Centroids of metal clusters are located in the (101) plane in these layers (Figure 5). 

### 3.3. Luminescent Properties

**Figure 6:**
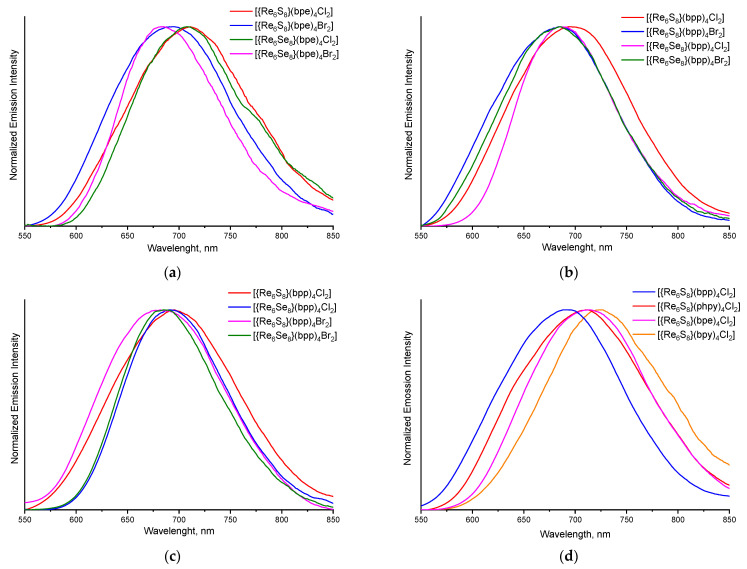
Normalized emission spectra of compounds **1**–**4** (**a**) and **5**–**8** (**b**) in the solid state, **5–8** in deaerated DMSO solution (**c**), as well as normalized solid-state emission spectra of a series of {Re_6_S_8_}^2+^-based complexes trans-[Re_6_S_8_L_4_Cl_2_] with L = bpp, phpy, bpe, and bpy (**d**).

Compounds **1**–**8** show broadband emission in the red region (Figure 6 and Figure S4, Supplementary Material). The spectroscopic and photophysical parameters of all investigated to date cluster complexes with the general formula *trans*-[Re_6_Q_8_L_4_X_2_] (Q = S or Se; X = Cl or Br; L = bpy, phpy, bpe or bpp) are summarized in Table 1. The Figures S5 and S6 show graphs with combined absorption and luminescence spectra for soluble complexes **5**–**8.** One can see that ligand type have a major influence on the emission parameters. Clusters with bpy and bpe ligands demonstrate small emission quantum yields (*Φ*_em_) and lifetimes (*τ*_em_). However, clusters with L = bpp or 4-phenylpyridine (phpy) are characterized by more than one order of magnitude higher *Φ*_em_ and *τ*_em_ values. This fact is in agreement with the previously published assumptions about luminescence quenching through the terminal heteroatom of the conjugated π-system [34]. Emission maximum wavelength (λ_em_) is another parameter, which can be controlled by the nature of apical organic ligand (Figure 6d). The emission maximum of corresponding clusters is shifted to a shorter wavelengths in the row bpy → phpy → bpe → bpp. In a recently published work from Yoshimura et al. [35], these shifts of λ_em_ were correlated with a different nature of the excited states, which, in its turn, depends on the structure of the lowest unoccupied molecular orbitals of the cluster complexes. 

### 3.4. DFT Calculations

**Figure 7:**
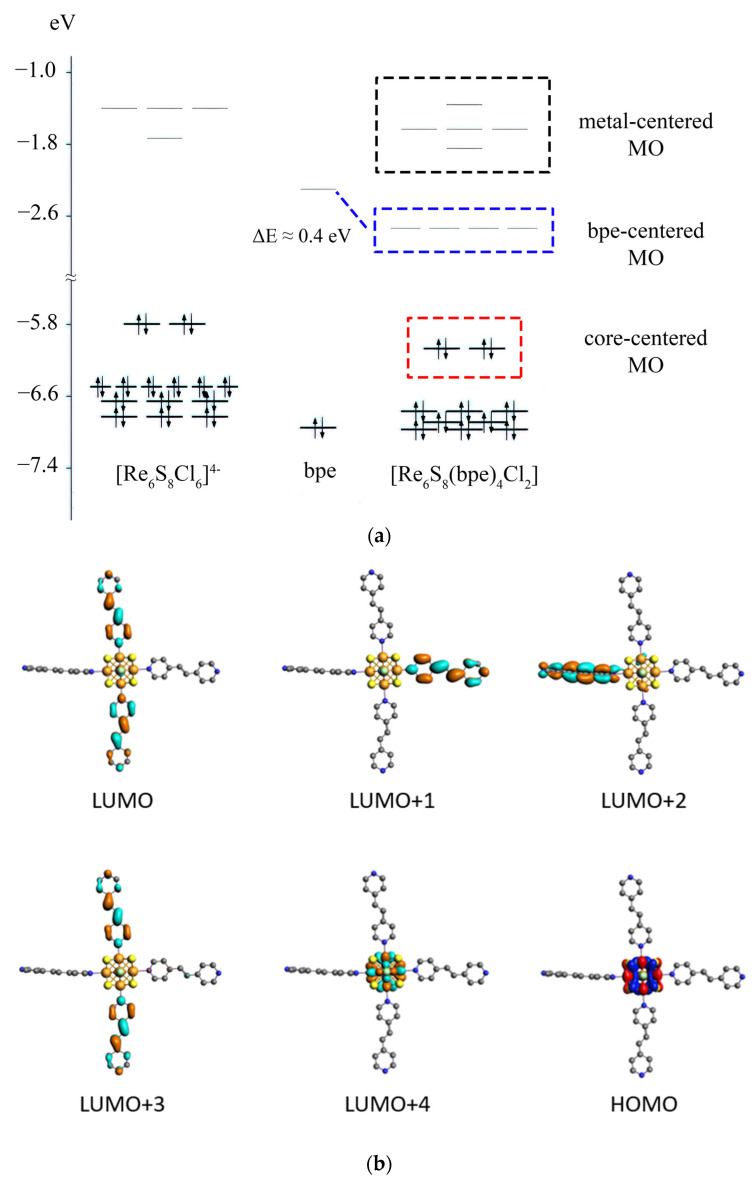
Energy levels diagram for the [Re_6_S_8_(bpe)_4_Cl_2_] cluster complex in comparison with energy levels of [Re_6_S_8_Cl_6_]^4–^ cluster anion and bpe molecule (**a**); molecular orbitals of the [Re_6_S_8_(bpe)_4_Cl_2_] cluster anion in near frontier region (**b**). Isosurface isovalue is 0.03 a.u.

The energy levels diagram and structure of molecular orbitals for the *trans*-[Re_6_S_8_(bpe)_4_Cl_2_] (**1**) and *trans*-[Re_6_S_8_(bpp)_4_Cl_2_] (**5**) clusters were calculated by DFT calculations. One can see that the compound **1** demonstrates the energy levels diagram similar to one found for other octahedral clusters with redox-active ligands such as pyrazine and bpy (Figure 7) [13,14]. While the “parent” cluster [Re_6_S_8_Cl_6_]^4–^ has the core-centered frontier molecular orbitals (MOs) with a large energy gap of 4.05 eV, cluster **1** demonstrates a series of four unoccupied MOs (LUMO-LUMO+3), which are localized between the core-centered HOMO and LUMO+4. These unoccupied orbitals are composed almost completely of π* orbitals of bpe and lie at lower energies than π* orbitals of free bpe molecule. The corresponding negative shift is about 0.4 eV, which is much less than the 0.8 eV shift for the π* orbital of the bpy molecule in the case of *trans*-[Re_6_Q_8_(bpy)_4_X_2_] cluster complexes. The LUMO-LUMO+3 are almost degenerated in energy, and the HOMO-LUMO gap is 3.30 eV for the *trans*-[Re_6_S_8_(bpe)_4_Cl_2_] cluster. The smaller energy gap between LUMO+3 and LUMO+4 is 0.89 eV. Therefore, the energy gap between core-centered orbitals HOMO and LUMO+4 remains almost unchanged in comparison with [Re_6_S_8_Cl_6_]^4-^ cluster anion.

**Figure 8:**
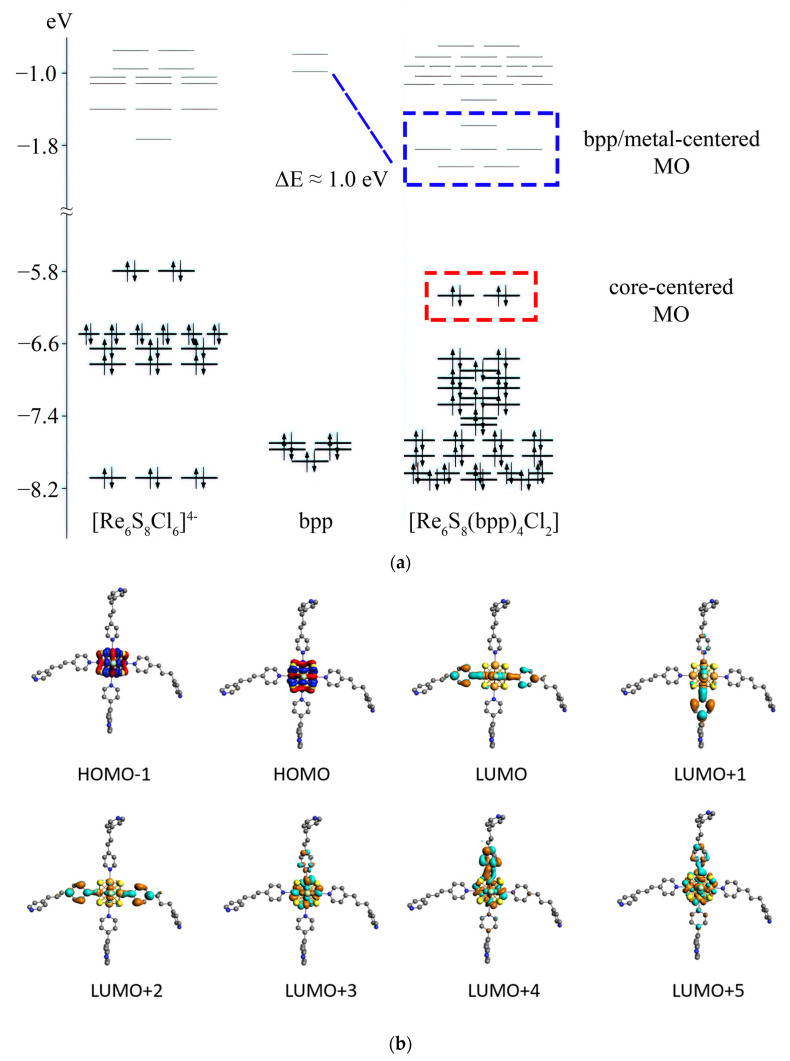
Energy levels diagram for the [Re_6_S_8_(bpp)_4_Cl_2_] cluster in comparison with energy levels of [Re_6_S_8_Cl_6_]^4–^ cluster anion and bpp molecule (**a**); molecular orbitals of the [Re_6_S_8_(bpp)_4_Cl_2_] cluster anion in near frontier region (**b**). Isosurface isovalue is 0.03 a.u.

In the case of *trans*-[Re_6_S_8_(bpp)_4_Cl_2_] cluster, a completely different picture is observed. Several lowest unoccupied orbitals are localized both on atoms of the coordinated pyridyne ring and atoms of the cluster core. Thus, frontier unoccupied orbitals in this compound have a mixed metal-ligand character, while HOMO and underlying orbitals are metal-centered (Figure 8). The negative shift of the π* orbitals of bpp in comparison with the free molecule is about 1 eV. The HOMO-LUMO gap for this cluster complex is 4.00 eV. 

### 3.5. Electrochemistry

**Figure 9:**
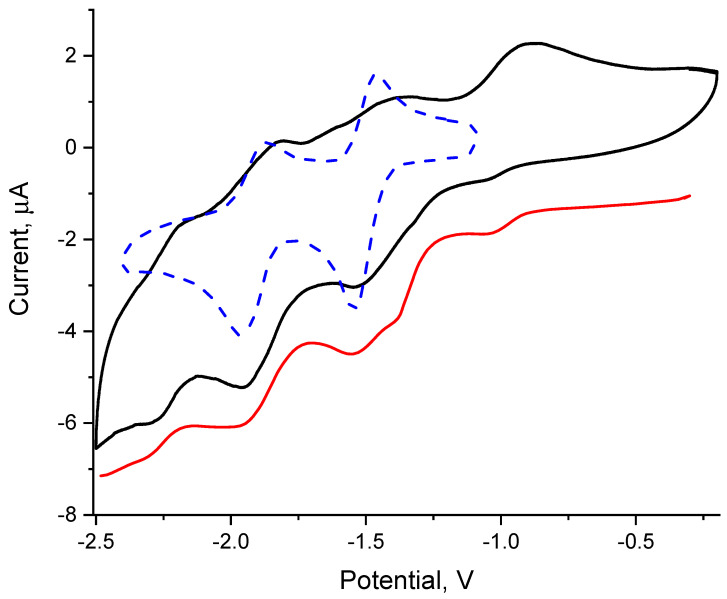
CV for *trans*-[Re_6_S_8_(bpe)_4_Cl_2_] (solid black) and free bpe (dotted blue). Square-wave voltammetry for *trans*-[Re_6_S_8_(bpe)_4_Cl_2_] (solid red). All measurements were carried out in DMSO, electrolyte: 0.1 M Bu_4_NBF_4_, CV scan rate: 100 mV/s.

Free bpe is capable of two reversible reduction processes in DMSO solution with E_1/2_ of −1.50 and −1.95 V (Figure 9). As in the case of 4,4ʹ-bpyridine, these reductions are associated with the generation of anion-radical and dianionic species. The coordination of bpe to the {Re_6_S_8_}^2+^ core led to appearance of a complex pattern of reduction processes. The potential of the first reduction process has shifted to the anodic area in comparison with the first reduction of the free molecule. This indicates a negative shift of the ligand’s molecular orbital levels on the energy scale. The series of redox transitions is lengthened up to potential values of about –2.25 V. It can be seen that some of the reduction half-waves have shoulders, which may indicate the presence of spatially unresolved processes. To show them more clearly, we used the method of square-wave voltammetry (Figure 9). This curve clearly shows the existence of several reduction processes. They are grouped in pairs and passing at potentials of −0.9 … −1.3 V, −1.4 … −1.8 V, and −1.9 … −2.2 V. A similar pattern was earlier observed for clusters with bpy ligand. In addition, the splitting of processes both in the case of clusters with bpy and bpe indicates the possibility of intramolecular charge transfer. Small differences in the positions of reduction processes are observed for cluster complexes with different inner chalcogenide and/or apical halide ligands. The Bpp ligand does not exhibit electrochemical activity in the potential window of DMSO or acetonitrile. For cluster complexes **5**–**8**, a series of spatially unresolved irreversible reduction processes appeared on the CV curves (Figure S7, Supplementary Material).

Scheme S1 represents a comparison between the first reduction potential values for [Re_6_Q_8_L_4_X_2_] cluster complexes with those for free ligands L in DMSO solution. It is worth noting that, for clusters with apical bpy and bpe molecules, the direction of the potential change with a decrease in the total electronegativity of the ligands is opposite. Specifically, for L = bpe the maximum potential is observed for cluster with Q = S, X = Cl, while in the case of L = bpy the maximum potential is observed for cluster with Q = Se, X = Br. The maximum negative shift value in the case of bpe ligands is 0.53 V, whereas in the case of clusters with bpy, the maximum shift was 1.04 V. Coordination of organic molecule to the cluster core causes significantly more pronounced effect on the position of π* orbitals in comparison with the effect of changes in the composition of the cluster core. The shift of the first reduction process strongly depends on the nature of the organic ligand. Thus, for the bpp ligand, the reduction potential is shifted by more than 1.1 V. For bpy ligands, this range is 0.84–1.04 V. For bpe ligans, the potential values are shifted by only 0.41–0.53 V. These values are in good agreement with those obtained from the DFT calculations. A characteristic feature of all the compounds **1**–**8** is the absence of the reversible oxidative process {Re_6_Q_8_}^2+/3+^ (24e/23e). This process is observed in all clusters of this type with inorganic apical ligands [1]. It has recently been shown that the coordination of organic molecules to the cluster core causes a strong shift in the oxidation potential of the core to the positive region, and the magnitude of the shift depends on the pKa of the ligand and the number of coordinated ligands [11,35]. Apparently, for complexes with four organic ligands, the value of the oxidation potential lies outside the electrochemical window of the solvents used.

**Figure 10:**
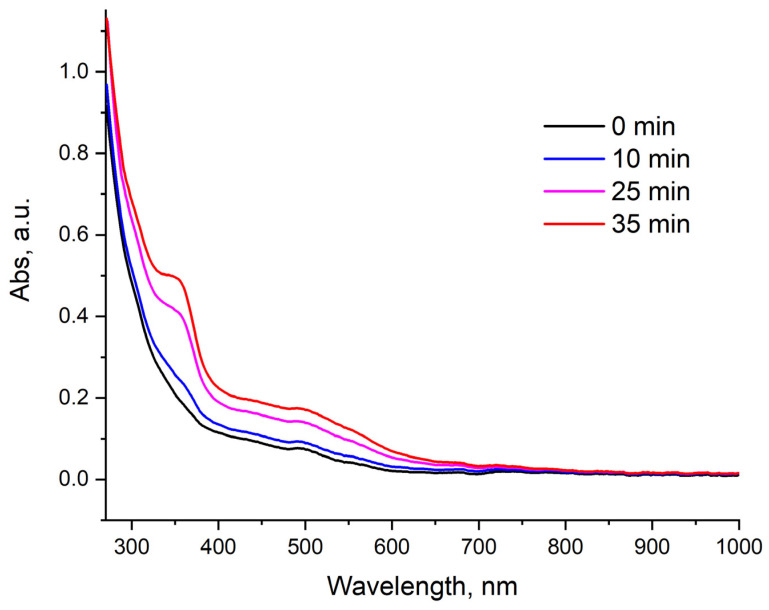
UV–vis spectroelectrochemical changes of a solution of **5** in DMSO. The experiment was carried out in a galvanostatic mode at a constant current value of −10 μA.

CV curves of clusters [Re_6_Q_8_(bpp)_4_X_2_] show a series of irreversible reduction processes. Sweep cycling showed that the concentration of the cluster in the near-electrode space does not decrease over many cycles (Figure S8). This indicates that the reported electrochemical transitions at low potentials are not accompanied by degradation of the compounds. In order to confirm the stability of the reduced forms of clusters, a spectroelectrochemical experiment was carried out with [Re_6_S_8_(bpp)_4_Cl_2_] since it has the highest solubility and the highest luminescence parameters. The change in the UV-vis spectrum is shown in Figure 10. The spectrum demonstrates appearance of the strong absorption band with a maximum at about 350 nm and an increase in absorption in the region of 400–600 nm during oxidation. After the applied potential was turned off, the profile of the UV-vis spectrum returned to the spectrum of the original compound. Therefore, we can assume that the reduction transitions are reversible and do not lead to a significant change in the geometry of the cluster core. The products of these transformations can be presented by reduced forms of the [Re_6_S_8_(bpp)_4_Cl_2_] cluster. Investigation of these species is of great interest due to the mixed character of the lowest unoccupied orbitals, which has been discussed above. 

## 4. Conclusions

In this work, study of the influence of coordinated N-donor aromatic molecules on the properties of octahedral rhenium cluster complexes was extended. Eight new octahedral rhenium cluster complexes with the general formula *trans*-[Re_6_Q_8_L_4_X_2_] (Q = S or Se; L = 1,2-Bis(4-pyridyl)ethylene (bpe) or 1,3-Bis(4-pyridyl)propane (bpp); X = Cl or Br) were synthesized and investigated. The bpe and bpp ligands represent two fundamentally different types of organic moieties: while bpe is a ligand with a conjugated aromatic system, bpp has independent aromatic systems of the two pyridine rings. The *trans*-[Re_6_Q_8_(bpe)_4_X_2_] clusters have the electronic structures similar to the structures of previously reported clusters with 4,4′-bipyridine, 4-phenylpyridine and pyrazine, that is, with redox-active ligands. In all these compounds, several lowest unoccupied molecular orbitals are essentially composed of π* orbitals of aromatic molecules, and the number of the orbitals is equal to the number of coordinated organic ligands. Moreover, a set of these orbitals, which are almost degenerated in energy, is separated from core-centered HOMO and overlying unoccupied orbitals by large energy gaps. On the contrary, the *trans*-[Re_6_Q_8_(bpp)_4_X_2_] clusters show the electronic structure with unoccupied orbitals having significant contribution from atoms of cluster core as well as organic molecules. Due to these differences, compounds with bpe and bpp ligands show very different electrochemical and luminescent properties. While the *trans*-[Re_6_Q_8_(bpe)_4_X_2_] clusters in solutions show multiple quasi-reversible electrochemical transitions associated with reduction of organic ligands, the *trans*-[Re_6_Q_8_(bpp)_4_X_2_] complexes show multielectron irreversible reduction processes. The emission lifetimes *τ*_em_ and quantum yields *Φ*_em_ for complexes with the bpp ligand exceed those for complexes with the bpe ligand by more than an order of magnitude. According to earlier studies, this is a result of the core-centered emissive excited triplet states of the complexes with bpp ligands.

## Figures and Tables

**Table 1:** Spectroscopic and photophysical parameters of *trans*-[Re_6_Q_8_L_4_X_2_] complexes (L = bpp, bpe, phpy, bpy). For all compounds except *trans*-[Re_6_Q_8_(bpp)_4_X_2_] measurements were carried out in solid state only due to low solubility. For compounds *trans*-[Re_6_Q_8_(bpp)_4_X_2_] measurements were also carried out in dearated DMSO solution.

	λ_em_, nm	*Φ*_em_	*τ*_em_, μs
[Re_6_S_8_(bpe)_4_Cl_2_] (**1**)	710	<0.005	0.57
[Re_6_S_8_(bpe)_4_Br_2_] (**2**)	690	<0.005	0.44
[Re_6_Se_8_(bpe)_4_Cl_2_] (**3**)	705	<0.005	n/a
[Re_6_Se_8_(bpe)_4_Br_2_] (**4**)	685	<0.005	n/a
[Re_6_S_8_(bpp)_4_Cl_2_] (**5**)	695 (solid) 695 (solution)	0.04 (solid) 0.05 (solution)	6.38 (solid) 6.80 (solution)
[Re_6_S_8_(bpp)_4_Br_2_] (**6**)	685 (solid) 682 (solution)	0.01 (solid) 0.02 (solution)	2.30 (solid) 7.10 (solution)
[Re_6_Se_8_(bpp)_4_Cl_2_] (**7**)	690 (solid) 692 (solution)	0.02 (solid) 0.02 (solution)	4.54 (solid) 4.71 (solution)
[Re_6_Se_8_(bpp)_4_Br_2_] (**8**)	685 (solid) 685 (solution)	0.005 (solid) 0.02 (solution)	3.48 (solid) n/a (solution)
[Re_6_S_8_(phpy)_4_Cl_2_]	708	0.028	4.3
[Re_6_S_8_(phpy)_4_Br_2_]	715	0.013	4.5
[Re_6_Se_8_(phpy)_4_Cl_2_]	708	0.015	3.8
[Re_6_Se_8_(phpy)_4_Br_2_]	718	0.013	4.0
[Re_6_S_8_(bpy)_4_Cl_2_]	725	<0.005	n/a
[Re_6_S_8_(bpy)_4_Br_2_]	730	<0.005	n/a
[Re_6_Se_8_(bpy)_4_Cl_2_]	725	0.02	n/a
[Re_6_Se_8_(bpy)_4_Br_2_]	730	0.01	n/a

## Data Availability

The XRD data were deposited with the Cambridge Crystallographic Data Centre (numbers 2212901 and 2212902) and can be requested from the authors or at http://www.ccdc.cam.ac.uk/conts/retrieving.html (access on 13 October 2022).

## Supplementary Materials

The following supporting information can be downloaded at: https://www.mdpi.com/article/10.3390/molecules27227874/s1, Table S1: Crystallographic data and structure refinement for compounds **1** and **2**; Table S2: Calculated atomic coordinates of optimized geometry of [Re_6_S_8_(bpe)_4_Cl_2_] cluster; Table S3: Calculated atomic coordinates of optimized geometry of [Re_6_S_8_(bpp)_4_Cl_2_] cluster; Figure S1: ^1^H NMR spectra for *trans*-[Re_6_Q_8_(bpe)_4_X_2_] (a) and *trans*-[Re_6_Q_8_(bpp)_4_X_2_] (b) cluster complexes; Figure S2: Overlap of bpe ligands between adjacent cluster fragments in the structure of compound **1·solv**; Figure S3: Overlap of bpe ligands between adjacent cluster fragments in the structure of compound **2·solv**; Figure S4: The [Re_6_S_8_(bpp)_4_Cl_2_] cluster under daylight and 365 nm lamp; Figure S5: Absorption and emission spectra of DMSO solutions of compounds **5** (black lines) and **6** (red lines); Figure S6: Absorption and emission spectra of DMSO solutions of compounds **7** (black lines) and **8** (red lines); Figure S7: CV in the negative region of the potential for *trans*-[Re_6_Q_8_(bpp)_4_X_2_] cluster complexes; Scheme S1: Dependence of the first reduction potential value for free bpe and [Re_6_Q_8_(bpe)_4_X_2_] (Q = S, X = Cl, Br; Q = Se, X = Cl) cluster complexes, free bpy and [Re_6_Q_8_(bpy)_4_X_2_] (Q = S, Se, X = Cl, Br) cluster complexes and free bpp and [Re_6_Q_8_(bpp)_4_X_2_] (Q = S, Se, X = Cl, Br); Figure S8: Cycling in the negative region of the potential for *trans*-[Re_6_S_8_(bpp)_4_Cl_2_] cluster complex. Electrolyte: 0.1 M Bu_4_NBF_4_, scan rate: 100 mV/s.

## Sample Availability

Samples of the compounds **1**–**8** are available from the authors.
