# Supplementary material for: Octahedral Rhenium Cluster Complexes with 1,2-Bis(4-pyridyl)ethylene and 1,3-Bis(4-pyridyl)propane as Apical Ligands
Source: Molecules. 2022 Nov 15;27(22):7874. doi: 10.3390/molecules27227874 (PMC9699422; doi:10.3390/molecules27227874)
Supplement: Supplementary file 1 [file molecules-27-07874-s001.zip › molecules-2000285-supplementary.pdf]

Supporting information for

Octahedral Rhenium Cluster Complexes with 1,2-Bis (4-pyridyl)Ethylene and 1,3-Bis(4-pyridyl)Propane as Apical Ligands

Anton A. Ulantikov, Konstantin A. Brylev, Taisiya S. Sukhikh, Yuri V. Mironov, Viktoria K. Muravieva and Yakov M. Gayfulin *

Nikolaev Institute of Inorganic Chemistry SB RAS, 3, academician Lavrentiev Ave., 630090 Novosibirsk, Russia

* Correspondence: gayfulin@niic.nsc.ru

Table S1. Crystallographic data and structure refinement for compounds **1** and **2.**

Compound	1·solv	2·solv
Empirical formula	$C_{81}H_{67.5}Cl_2N_{13.5}Re_6S_8$	$C_{84}H_{70}Br_2N_{14}Re_6S_8$
Formula weight	2674.56	2809.04
Crystal system, space group	triclinic, *P*-1	triclinic, *P*-1
a/Å	12.2441(5)	12.5294(5)
b/Å	23.6588(11)	16.9217(6)
c/Å	30.2956(12)	21.9328(9)
α/°	71.370(2)	96.3550(10)
β/°	82.2110(10)	104.830(2)
γ/°	77.6590(10)	107.1350(10)
Volume/Å^3	8102.6(6)	4207.7(3)
Z	4	2
ρ_{calc}g/cm^3	2.192	2.217
μ/mm^{-1}	9.254	9.798
F(000)	5040	2640
Crystal size/mm^3	0.12 × 0.03 × 0.03	0.04x0.03x0.03
2Θ range for data collection/°	1.445 to 26.372	1.785 to 24.406
Index ranges	$-15 \leq h \leq 15$, $-29 \leq k \leq 29$, $-37 \leq l \leq 37$	$-14 \leq h \leq 14$, $-19 \leq k \leq 19$, $-25 \leq l \leq 25$
Reflections collected	182820	63110
Independent reflections	33124 R_{int} = 0.0566, R_{sigma} = 0.0463	13859 R_{int} = 0.0677, R_{sigma} = 0.0577
Goodness-of-fit on F^2	1.034	1.016
Final R indexes [I>=2σ (I)]	R_1 = 0.0361, wR_2 = 0.0726	R_1 = 0.0326, wR_2 = 0.0576
Final R indexes [all data]	R_1 = 0.0570, wR_2 = 0.0842	R_1 = 0.0582, wR_2 = 0.0674
Largest diff. peak/hole / e Å^{-3}	2.547/-1.246	0.816/-0.740

Table S2. Calculated atomic coordinates of optimized geometry of $[Re_6S_8(bpe)_4Cl_2]$ cluster.

Atom	x	y	z	Atom	x	y	z
Re	12.48353	36.97181	13.66467	C	23.19974	34.73884	16.87226
Re	14.74021	36.10722	12.71843	C	22.63689	32.43802	17.13887
Re	12.58020	34.73907	12.34463	C	10.22253	25.72067	14.28762
Re	11.45157	36.81467	11.29253	C	8.11127	26.09981	13.24833
Re	13.61592	38.18029	11.66644	C	3.00942	39.45409	7.75590
Re	13.70257	35.94929	10.34325	C	3.80416	38.85050	5.59156
S	11.64040	34.71889	10.13380	C	14.46380	47.49327	8.89985
S	14.54456	38.20185	13.88174	C	16.58514	47.06107	9.89720
S	13.58373	35.00238	14.51216	C	24.39960	34.45947	17.50776
S	10.52326	35.65458	13.18026	C	23.86582	32.26076	17.76164
S	11.49117	38.86295	12.55004	C	9.80695	24.41986	14.52596
S	15.66978	37.25793	10.82625	C	7.78934	24.77903	13.53397
S	14.70820	34.06207	11.45706	C	1.83407	39.86537	7.14615
S	12.60836	37.92308	9.50185	C	2.59041	39.28895	5.07759
Cl	11.66746	37.64932	15.86011	C	14.90748	48.78216	8.63222
Cl	14.50296	35.27337	8.14158	C	16.93290	48.36624	9.58543
N	9.46989	37.27155	10.43188	H	16.26460	33.71593	14.01937
N	14.24414	40.25519	11.24789	H	17.40540	37.61914	13.26064
N	16.72536	35.69123	13.59619	H	10.07387	32.92339	11.95438
N	11.96539	32.65904	12.75882	H	13.78973	32.17118	13.61077
N	24.75376	33.24430	17.95602	H	12.42146	40.76241	10.40106
N	8.61048	23.92913	14.16396	H	8.68419	37.85737	12.25807
N	1.60118	39.79456	5.82555	H	16.13328	39.97229	12.04893
N	16.12358	49.23688	8.96081	H	10.03736	36.72356	8.51511
C	17.03179	34.47859	14.10471	H	6.01859	38.10226	6.73335
C	17.66412	36.65312	13.68137	H	13.81161	45.04383	9.49557
C	10.73624	32.20705	12.42953	H	19.60036	37.27042	14.31259
C	12.80677	31.79132	13.35292	H	9.31413	30.63840	12.37426
C	9.22658	37.12993	9.11113	H	13.18832	29.83989	14.11117
C	8.47495	37.75968	11.19774	H	7.92463	37.33945	7.45443
C	13.41042	41.13333	10.64981	H	21.13951	35.91026	15.46815
C	15.47712	40.69091	11.56907	H	11.64785	28.06248	14.09492
C	10.32099	30.91529	12.67244	H	6.49571	38.52614	11.35519
C	18.23806	34.19894	14.70951	H	13.03376	43.06754	9.87605
C	18.89284	36.44599	14.27368	H	5.01411	38.81816	9.54990
C	12.45870	30.48532	13.62864	H	23.00025	35.75713	16.54816
C	8.02956	37.48291	8.52563	H	8.89520	28.54836	12.81421
C	7.25102	38.13177	10.68000	H	21.96521	31.59022	17.01801
C	13.76936	42.43148	10.35923	H	16.55463	44.43460	10.74494
C	15.90456	41.97705	11.30932	H	11.21117	26.02683	14.62026
C	19.22090	35.19640	14.82143	H	20.35755	32.98412	15.99781
C	11.18673	29.99850	13.29146	H	7.39108	26.73192	12.73218
C	6.99062	38.01721	9.30578	H	16.91428	42.26081	11.59530
C	15.05250	42.90077	10.68504	H	18.39446	33.19618	15.09571

C	20.51417	35.01767	15.45902	H	3.10487	39.55356	8.83410
C	10.85340	28.61774	13.59646	H	4.56241	38.45179	4.92018
C	5.70477	38.45525	8.78888	H	13.46046	47.19461	8.60195
C	15.53165	44.24664	10.41947	H	17.31000	46.43056	10.40583
C	20.97759	33.88080	16.01822	H	25.12271	35.25988	17.66911
C	9.68908	27.99635	13.31827	H	24.15100	31.27282	18.12558
C	5.32565	38.46310	7.49402	H	10.47426	23.72672	15.03956
C	14.83377	45.23536	9.82329	H	6.81660	24.38286	13.23953
C	22.26964	33.70716	16.66861	H	1.02805	40.27825	7.75398
C	9.36165	26.61043	13.62561	H	2.40403	39.23088	4.00440
C	4.04584	38.92381	6.97123	H	14.24798	49.48771	8.12524
C	15.31067	46.58470	9.55118	H	17.92274	48.73825	9.85270

Table S3. Calculated atomic coordinates of optimized geometry of [$Re_6S_8(bpp)_4Cl_2$] cluster.

Atom	x	y	z	Atom	x	y	z
Re	13.46401	37.02279	12.71433	H	3.94766	37.23189	9.66184
Re	15.80827	36.21593	11.94157	H	2.10492	37.51374	5.77645
Re	13.71400	34.83455	11.34222	H	13.66114	47.98420	12.79064
Re	12.64486	36.91327	10.25605	H	17.96577	48.00653	12.81438
Re	14.73611	38.31375	10.86386	H	22.33367	29.82053	19.52444
Re	14.99361	36.12482	9.48653	H	24.01807	28.57480	15.96684
S	11.57605	35.69816	12.02701	H	8.24378	31.35730	18.69408
S	14.51772	35.04829	13.59697	H	7.61699	27.31480	19.24725
S	16.87715	37.46495	10.18547	H	1.71450	36.80999	10.59098
S	15.91665	34.20905	10.62134	H	-0.06092	37.07801	6.87389
S	12.99456	34.85738	9.05333	H	13.73073	49.24351	14.91639
S	12.52150	38.92330	11.56663	H	17.85935	49.26567	14.93927
S	15.48178	38.24853	13.15504	H	17.13427	33.76338	13.26356
S	13.92419	38.08768	8.60662	H	18.48833	37.63759	12.75936
Cl	12.45661	37.62152	14.85232	C	17.92939	34.48426	13.42026
Cl	16.01332	35.54106	7.34913	C	18.68550	36.64013	13.14006
N	15.27156	40.48088	10.62472	C	11.80772	32.36255	11.36120
N	13.04776	32.75819	11.70680	C	13.78084	31.92304	12.46702
N	23.18100	29.07359	17.79151	C	10.37217	38.48500	8.75973
N	7.86258	29.36430	19.08658	C	9.88919	36.24200	8.96706
N	0.70519	36.92085	8.79118	C	15.68171	41.21988	11.67387
N	15.79419	49.31956	15.04341	C	15.06759	41.13945	9.46367
N	17.70958	35.72764	12.95786	C	19.09564	34.11633	14.06459
N	10.72631	37.26925	9.21394	C	19.87370	36.33874	13.77563
H	11.24967	33.03377	10.71667	C	11.24495	31.18925	11.82444
H	14.79076	32.24468	12.69961	C	13.27855	30.73682	12.96645
H	11.05971	39.29775	8.97274	C	9.20398	38.70392	8.04933
H	10.19133	35.27217	9.34705	C	8.71759	36.38960	8.25484
H	15.85421	40.68610	12.60116	C	15.86215	42.58984	11.61351
H	14.74626	40.53891	8.62007	C	15.23140	42.50468	9.33522
H	19.18651	33.09008	14.40625	C	20.11412	35.04869	14.26310
H	20.61742	37.12416	13.89085	C	11.96345	30.35947	12.68815
H	10.22751	30.94306	11.52962	C	8.34366	37.64338	7.76337
H	13.91397	30.12358	13.60107	C	15.62376	43.28377	10.42788
H	8.98171	39.71117	7.70506	C	21.40820	34.72623	14.95438
H	8.10322	35.51035	8.07563	C	11.32367	29.18530	13.35957
H	16.17958	43.10028	12.51791	C	7.08256	37.81671	6.97259
H	15.03249	42.96209	8.36846	C	15.77790	44.77131	10.29244
H	21.55408	35.46788	15.75354	C	21.54041	33.32143	15.52501
H	22.22382	34.91473	14.23989	C	10.60910	29.63497	14.64398
H	10.59896	28.71568	12.68264	C	5.81114	37.60576	7.80457
H	12.08433	28.43421	13.60555	C	15.77459	45.57076	11.58997
H	7.06782	38.81196	6.51285	C	22.88089	33.10681	16.23684
H	7.08926	37.08482	6.15229	C	9.95403	28.47857	15.38279

H	14.98177	45.13549	9.62865	C	4.56038	37.67961	6.94006
H	16.71609	44.96091	9.74759	C	15.82497	47.08173	11.33310
H	21.44254	32.57778	14.72284	C	23.00862	31.71536	16.78482
H	20.72593	33.12806	16.23611	C	9.24048	28.82089	16.66487
H	11.33713	30.13387	15.29791	C	3.24176	37.41632	7.61762
H	9.85632	30.38957	14.37986	C	15.81539	47.87544	12.60688
H	5.86259	36.62671	8.29869	C	22.56840	31.38852	18.06911
H	5.77392	38.36009	8.60148	C	23.53606	30.67167	16.02186
H	16.63386	45.29142	12.21327	C	9.06426	30.12268	17.13534
H	14.87287	45.33265	12.17033	C	8.70165	27.80031	17.45610
H	22.97643	33.83309	17.05436	C	3.09384	37.21113	8.98943
H	23.70049	33.30111	15.53287	C	2.06904	37.36561	6.85504
H	9.23098	27.98255	14.71782	C	14.62233	48.24202	13.23324
H	10.70855	27.71129	15.60932	C	16.99719	48.25453	13.24623
H	4.50218	38.66911	6.46221	C	22.67332	30.07778	18.51992
H	4.65441	36.96698	6.10742	C	23.60138	29.39111	16.55904
H	14.96434	47.37100	10.71585	C	8.38050	30.33803	18.32909
H	16.72971	47.32050	10.75914	C	8.03568	28.11298	18.63234
H	22.14873	32.15185	18.72284	C	1.82761	36.97153	9.51773
H	23.90289	30.85239	15.01240	C	0.85105	37.11978	7.47155
H	9.45016	30.98077	16.59102	C	14.66162	48.95120	14.42800
H	8.80118	26.75796	17.15613	C	16.93709	48.96344	14.44068

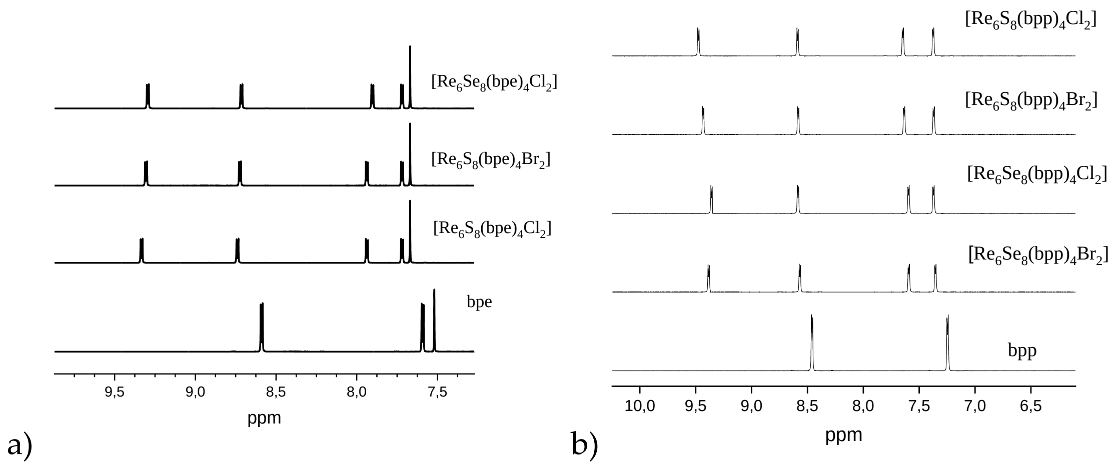

Figure S1. ^{1}H NMR spectra for *trans*-[$Re_6Q_8(bpe)_4X_2$] (a) and *trans*-[$Re_6Q_8(bpp)_4X_2$] (b) cluster complexes.

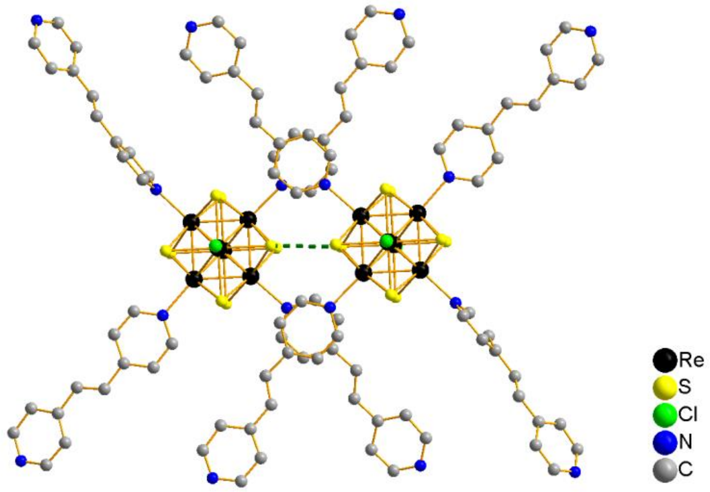

Figure S2. Overlap of bpe ligands between adjacent cluster fragments in the structure of compound **1·solv**. The short contact between the μ_3-S ligands is shown by the dotted line.

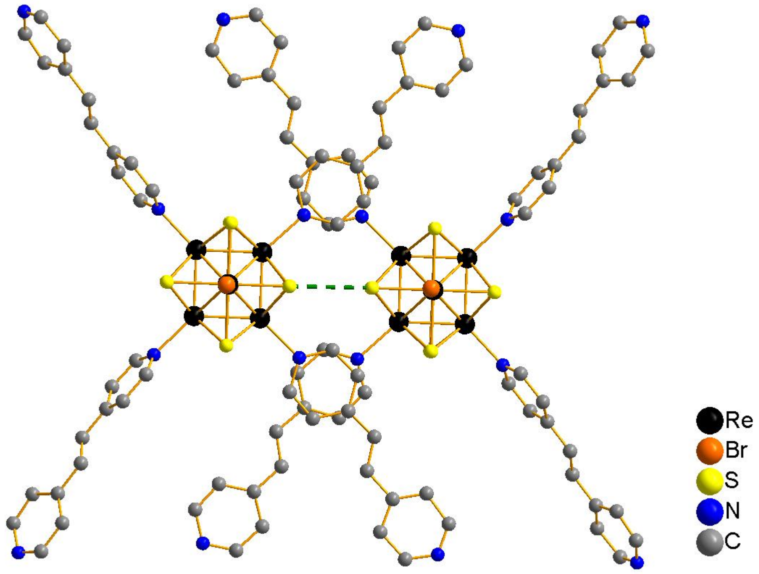

Figure S3. Overlap of bpe ligands between adjacent cluster fragments in the structure of compound **2·solv**. The short contact between the μ_3-S ligands is shown by the dotted line.

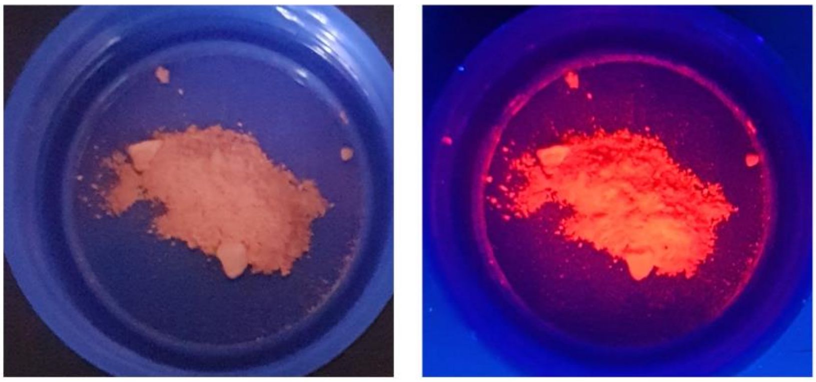

Figure S4. The $[Re_6S_8(bpp)_4Cl_2]$ cluster under daylight (left) and 365 nm lamp (right).

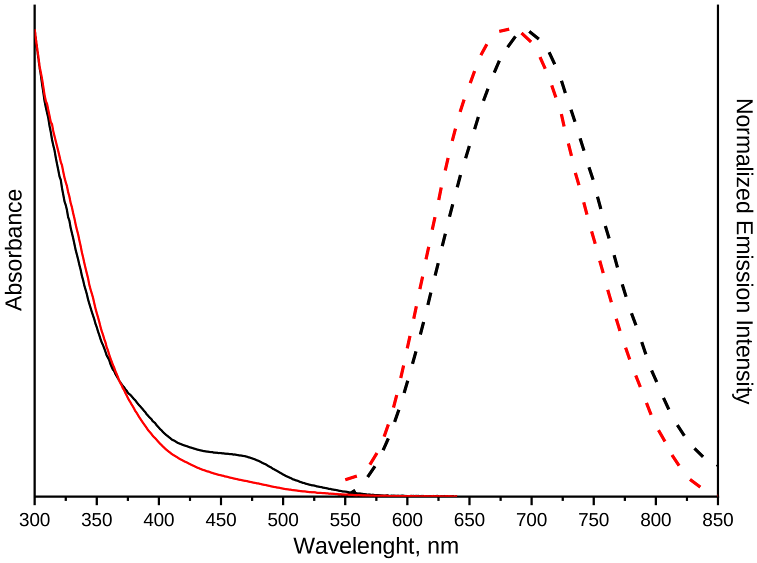

Figure S5. Absorption and emission spectra of DMSO solutions of compounds **5** (black lines) and **6** (red lines).

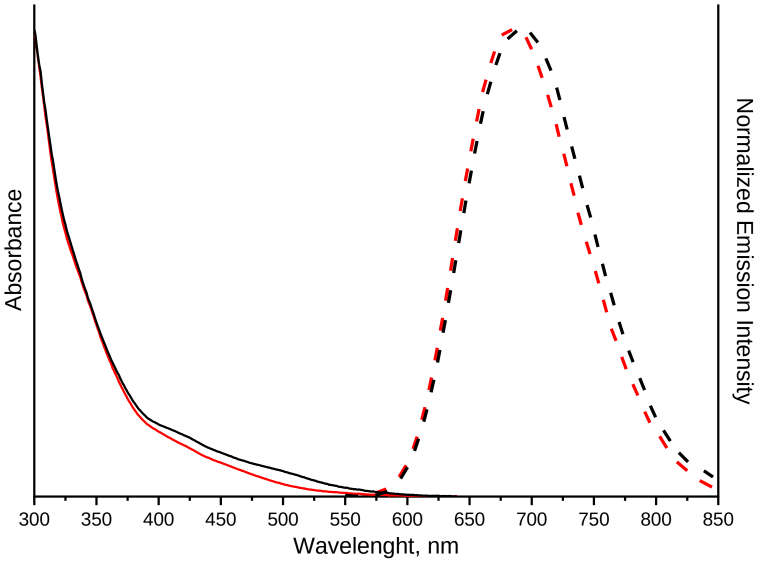

Figure S6. Absorption and emission spectra of DMSO solutions of compounds **7** (black lines) and **8** (red lines).

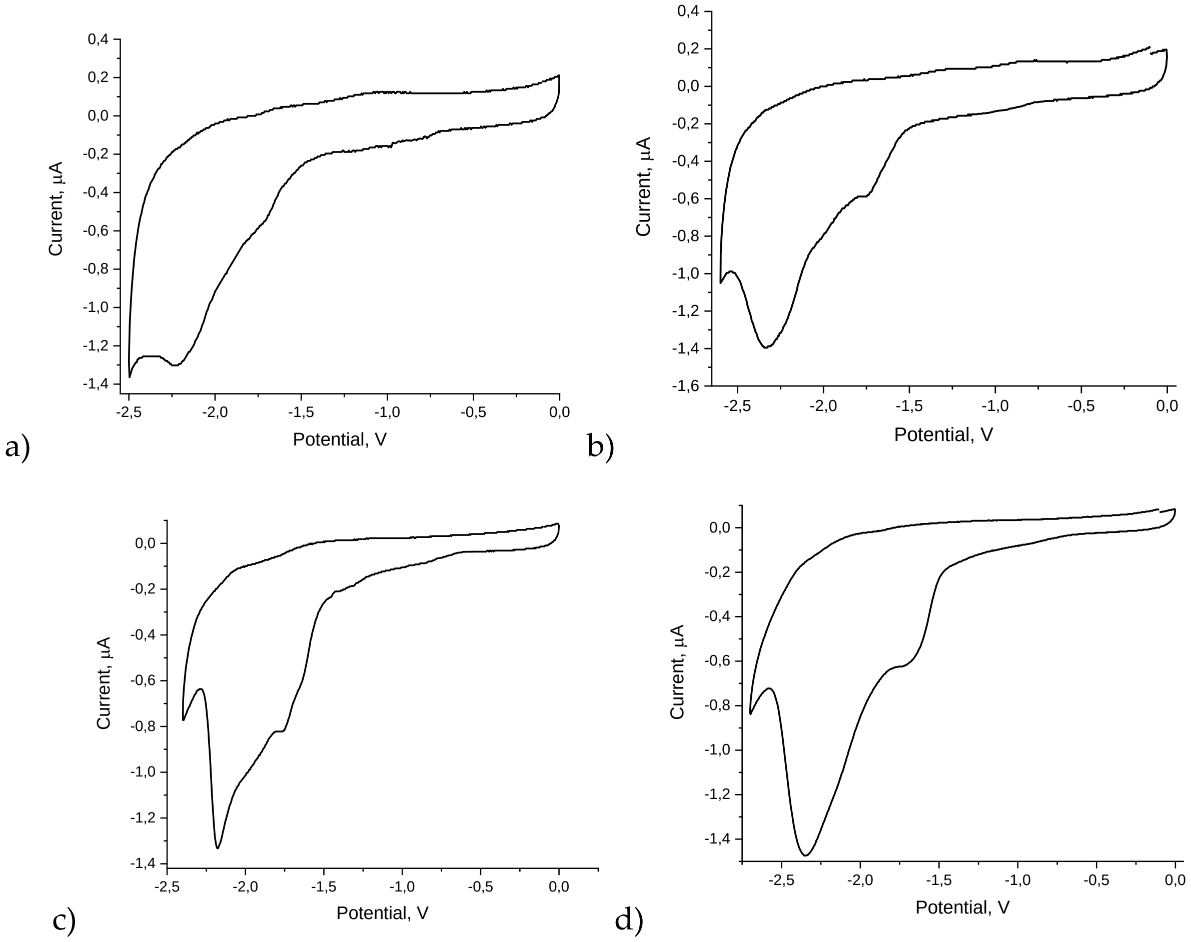

Figure S7. CV in the negative region of the potential for *trans*-[$Re_6Q_8(bpp)_4X_2$] (Q = S, X = Cl (a), Q = S, X = Br (b), Q = Se, X = Cl (c), Q = Se, X = Br (d)) cluster complexes. Electrolyte: 0.1 M Bu_4NBF_4, scan rate: 100 mV/s.

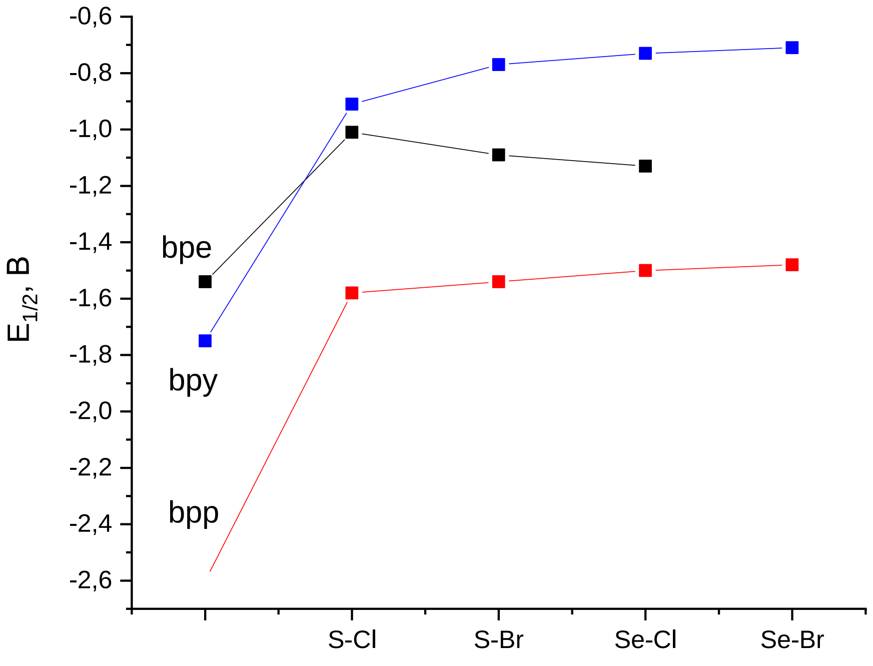

Scheme S1. Dependence of the first reduction potential value for free bpe and [$Re_6Q_8(bpe)_4X_2$] (Q = S, X = Cl, Br; Q = Se, X = Cl) cluster complexes (black), free bpy and [$Re_6Q_8(bpy)_4X_2$] (Q = S, Se, X = Cl, Br) cluster complexes (blue) and free bpp and [$Re_6Q_8(bpp)_4X_2$] (Q = S, Se, X = Cl, Br) (red).

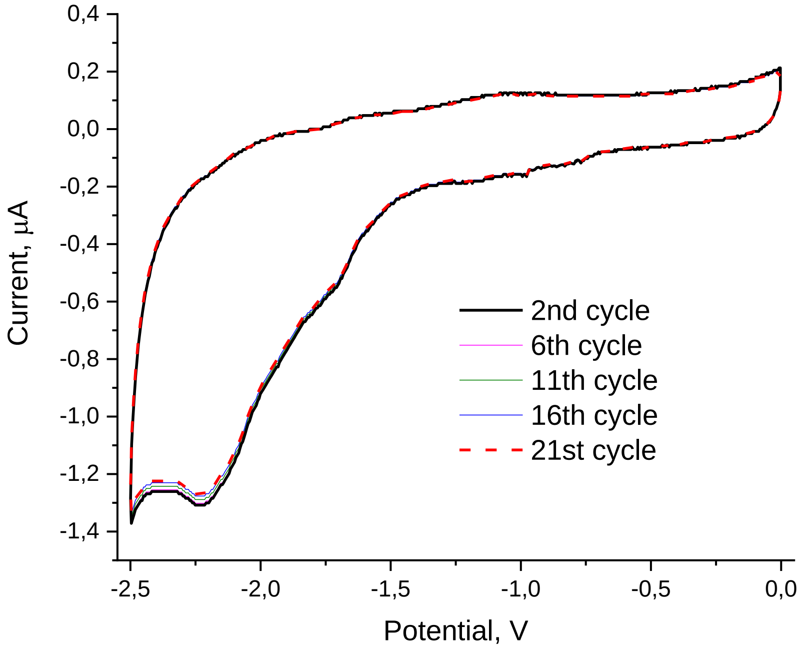

Figure S8. Cycling in the negative region of the potential for *trans*-[$Re_6S_8(bpp)_4Cl_2$] cluster complex. Electrolyte: 0.1 M Bu_4NBF_4, scan rate: 100 mV/s.
